# Functional validation to explore the protective role of miR-223 in *Staphylococcus aureus*-induced bovine mastitis

**DOI:** 10.1186/s40104-025-01152-6

**Published:** 2025-03-04

**Authors:** Xueqin Liu, Siyuan Mi, Gerile Dari, Siqian Chen, Jiuzhou Song, David E. MacHugh, Ying Yu

**Affiliations:** 1 College of Animal Science and Technology, China Agricultural University, Beijing, 100193 China; 2 UCD School of Agriculture and Food Science, University College Dublin, Dublin, D04 V1W8 Ireland; 3 Department of Animal and Avian Sciences, University of Maryland, College Park, MD USA; 4 UCD Conway Institute of Biomolecular and Biomedical Research, University College Dublin, Dublin, D04 V1W8 Ireland; 5 UCD Centre for One Health, University College Dublin, Belfield, Dublin, D04 V1W8 Ireland

**Keywords:** Bovine mastitis, Gene regulation, Mammary epithelial cells, MiR-223, *Staphylococcus aureus*

## Abstract

**Background:**

Mastitis caused by *Staphylococcus aureus* (*S. aureus*) is one of the most intractable problems for the dairy industry, causing significantly reduced milk yields and early slaughter of cows worldwide. MicroRNAs (miRNAs) can post-transcriptionally regulate gene expression and studies in recent years have shown the importance of miRNA-associated gene regulation in *S. aureus*-induced mastitis.

**Results:**

In this study, to investigate the role of miR-223 in mastitis, we performed experiments to overexpress and suppress miR-223 in an immortalized bovine mammary epithelial cell line (MAC-T) infected with *S. aureus*. Overexpression of miR-223 in MAC-T cells repressed cell apoptosis and necrosis induced by *S. aureus* infection, whereas suppression of miR-223 had the opposite effect. Transcriptome expression profiling with weighted gene co-expression network analysis (WGCNA) and gene set variation analysis (GSVA) showed that miR-223 affects apoptosis and inflammation-related pathways. Furthermore, differentially expressed (DE) genes were evaluated, and genes exhibiting contrasting expression trends in the miR-223 overexpressed and suppressed groups were assessed as potential target genes of miR-223. Potential target genes, including *CDC25B*, *PTPRF*, *DCTN1*, and *DPP9*, were observed to be associated with apoptosis and necroptosis. Finally, through integrative analysis of genome-wide association study (GWAS) data and the animal quantitative trait loci (QTL) database, we determined that target genes of miR-223 were significantly enriched in single-nucleotide polymorphisms (SNP) and QTLs related to somatic cell count (SCC) and mastitis.

**Conclusion:**

In summary, miR-223 has an inhibitory effect on *S. aureus*-induced cell apoptosis and necrosis by regulating *PTPRF*, *DCTN1*, and *DPP9*. These genes were significantly enriched in QTL regions associated with bovine mastitis resistance, underscoring their relevance in genetic regulation of disease resilience. Our findings provide critical genetic markers for enhancing mastitis resistance, particularly *S. aureus*-induced mastitis, through selective breeding. This work offers valuable insights for developing cattle with improved resistance to mastitis via targeted genetic selection.

**Supplementary Information:**

The online version contains supplementary material available at 10.1186/s40104-025-01152-6.

## Background

Bovine mastitis is a major challenge for the global dairy industry, leading to reduced milk production, poor milk quality, and substantial economic losses, as well as animal welfare concerns [1, 2]. The disease is typically triggered by bacterial pathogens such as *Staphylococcus aureus*, *Streptococcus agalactiae*, and *Escherichia coli* [3]. It is characterized by udder inflammation and milk composition changes, making it difficult to manage [4, 5]. Given the limitations of current diagnostic and treatment methods, innovative approaches are needed to alleviate the impact of mastitis on dairy production.

MicroRNAs (miRNAs) are important post-transcriptional regulators in eukaryotic cells, influencing gene expression by targeting mRNAs and affecting cellular response mechanisms [6–10]. Their dysregulation has been linked to various cancers, infectious diseases, and abnormal immune responses, including in bovine mastitis, where it affects disease progression and severity [11–15]. Although previous studies have implicated miR-223 in regulating immune responses to *S. aureus* mastitis, their focus has been limited to specific pathways or broader transcriptional profiles without fully elucidating miR-223’s precise role in infection severity [16, 17]. However, the precise mechanisms by which miR-223 modulate the immune response in bacterial-induced mastitis, particularly in infections caused by *S. aureus*, remain unclear.

Therefore, this study aims to investigate the functional role of miR-223 in *S. aureus* infections, particularly in modulating the immune response in *S. aureus*-induced mastitis. By elucidating these regulatory mechanisms, we hope to provide new insights into targeted diagnostic and therapeutic strategies for bovine mastitis. Not only does this study offer potential solutions to reduce the economic burden of mastitis on the dairy industry, but it also lays the groundwork for future miRNA-based disease resistance breeding strategies aimed at enhancing bovine health.

## Methods

### Establishment of stable MAC-T cell cultures

MAC-T cells were derived from an established clonal cell line, which was originally produced from primary bovine mammary alveolar cells [18]. For this study, MAC-T cells were revived from cryopreservation and cultured in Dulbecco's Modified Eagle Medium (DMEM, Gibco, Grand Island, NY, USA), supplemented with 10% foetal bovine serum (FBS, Gibco, Grand Island, NY, USA) and 1% antibiotics. The antibiotics used were a combination of penicillin, at a final concentration of 100 U/mL, and streptomycin, at 100 μg/mL (Invitrogen, Carlsbad, CA, USA). The culture was maintained at 37 °C in a humidified incubator with 5% CO_2_. The initial culture involved continuously culturing the MAC-T cells for three generations, with each generation lasting approximately 48 h, to establish a stable cell population. During this phase, the cells were carefully monitored to ensure optimal growth conditions and prevent contamination. After achieving stable growth, the cells were suspended in complete culture medium to obtain a uniform cell suspension.

### Transfection of MAC-T cells with bta-miR-223 mimics and inhibitors

The bta-miR-223 mimics and inhibitors used in this study were synthesized by Suzhou GenePharma Co., Ltd. (Suzhou, China). MAC-T cells were cultured in six-well plates until they reached a density of approximately 60%–70%. The miRNA inhibitors or mimics were then transfected into the cells using Lipofectamine 3000 (Lip3000, Thermo Fisher Scientific, Waltham, MA, USA) according to the manufacturer’s instructions. To prepare the transfection complexes, Lip3000 was diluted in optimized minimum essential medium (Opti-MEM), and the miRNA inhibitors or mimics were diluted separately in Opti-MEM to a final concentration of 20 μmol/L. The Lip3000-Opti-MEM solution was then combined with the diluted miRNA inhibitors or mimics, and the mixtures were incubated for 10–15 min to form transfection complexes. The transfection complexes were added to the MAC-T cells, ensuring an even distribution, and incubated at 37 °C and 5% CO_2_ for approximately 48 h. The transfection efficiency was monitored using an inverted fluorescence microscope, and quantitative real-time PCR (qPCR) was used to assess the efficiency of miRNA overexpression or inhibition.

### Preparation of *S. aureus* suspension and challenge of MAC-T cells

A *S. aureus* suspension was prepared by inoculating 1 g of tryptic soy broth (TSB) powder into 100 mL of TSB medium, followed by overnight incubation at 37 °C with shaking at 200 r/min. The bacterial suspension was then diluted in fresh TSB medium to achieve a final concentration of approximately 10^8^ CFU/mL.

After transfection with bta-miR-223 mimics, inhibitors, or their respective controls, MAC-T cells were divided into four groups: knockdown (KD), control knockdown (CKD), overexpression (OE), and control overexpression (COE). The transfection medium was carefully removed, and cells were washed twice with sterile phosphate-buffered saline (PBS) to remove any remaining medium. Subsequently, the *S. aureus* suspension was added to the MAC-T cells at a multiplicity of infection (MOI) of 10:1. The infected cells were incubated at 37 °C with 5% CO_2_ for the infection period, which was typically around 6 h. After incubation, the infected MAC-T cells were washed with sterile PBS to remove non-adherent bacteria. The cells were then collected for further analysis.

### Assessment of apoptosis and necrosis in MAC-T cells

Apoptosis and necrosis in MAC-T cells were assessed using the YO-PRO-1 (YP1)/Propidium Iodide (PI) Apoptosis and Necrosis Detection Kit (Beyotime Biotechnology, Shanghai, China). PI stains dead cells with compromised membranes, emitting red fluorescence, which reflects the number of necrotic cells. YP1, which emits green fluorescence, selectively enters early apoptotic cells, enabling detection of the early stages of programmed cell death. Together, these dyes can distinguish necrotic, apoptotic, and live cells in a sample.

MAC-T cells were harvested and washed twice with PBS. The cells were then resuspended in staining buffer from the kit at a concentration of 1 × 10^6^ cells/mL, suitable for analysis. A 5 µL volume of the YP1/PI staining solution was added to each 100 µL of cell suspensions. The resuspension was then gently mixed and incubated at 37 °C in the dark for 30 min to ensure proper staining. After incubation, the stained samples were analysed using fluorescence microscopy to differentiate between apoptotic and necrotic cells. The samples were also examined with a fluorescence plate reader to quantitatively assess the proportion of apoptotic and necrotic cells in each well.

### RNA isolation and mRNA sequencing analysis

Total RNA was extracted using the TRIzol reagent (Takara, Beijing, China) following the manufacturer’s instructions to ensure minimal RNA degradation. The RNA sequencing (RNA-seq) library construction and sequencing were performed by Novogene Co., Ltd. (Beijing, China). RNA samples were sequenced on the Illumina HiSeq 2500 platform to produce 150 bp paired-end reads.

The quality of the raw sequencing reads was evaluated using FastQC v0.11.8 [19] and NGS QC Toolkit v2.3.3 was used for quality control and filtering of the reads to remove low-quality reads and adapter sequences [20]. The filtered reads were aligned to the reference genome Bos_taurus.ARS-UCD1.2.107 [21] using Hisat2 v2.1.0 [22], which also generated genome indices. The Sequence Alignment/Map (SAM) files generated were then converted to Binary Alignment/Map (BAM) format using SAMtools v1.9 [23].

The featureCounts v1.6.3 program was used to count the aligned reads and map them to specific genomic features [24]. The DESeq2 v1.28.1 package [25] in R (version 4.3.0) [26] was employed for differential gene expression analysis, normalizing read counts and identifying differentially expressed genes. The statistical significance of the differentially expressed genes was determined using *P*-values adjusted for multiple testing with the Benjamini–Hochberg method (BH-*P*_adj._ < 0.05) [27].

### Prediction of bta-miR-223 target genes

The RNA22 v2, miRanda v3.3a, and TargetScan v7.2 software tools were used to predict the target genes of bta-miR-223 and the intersection of results from the three tools was used for subsequent analyses. RNA22 [28], miRanda [29], and TargetScan [30] predict miRNA binding sites and target genes using distinct methodologies. Used together, these tools provide a comprehensive methodology for studying miRNA-mRNA interactions and understanding gene regulation mechanisms.

### Functional analysis of transcriptomic data

Following basic analysis of the transcriptomic data, functional characterization was performed using weighted gene co-expression network analysis (WGCNA) with the WGCNA package v1.72.5 [31] in R (version 4.3.0) [26]. Modules were identified through hierarchical clustering, grouping genes based on their expression similarities. By correlating these modules with the experimental groups, the analysis identified gene networks where bta-miR-223 plays a role in host–pathogen interaction for *S. aureus*-induced mastitis.

Gene Set Variation Analysis (GSVA) [32] was performed using the GSVA R package v1.50.5. The GSVA algorithm calculates an enrichment score for each gene set in each sample as follows. For each sample, genes were ranked based on their expression levels. For each gene set, the enrichment score was computed by assessing whether the genes in the set are predominantly at the top or bottom of the ranked list. This was done using a kernel estimation of the cumulative distribution function. Enrichment scores were normalized to facilitate comparisons across samples. The GSVA scores were subjected to statistical analysis to identify significant differences between groups. To do this, differential enrichment analysis was performed using the limma package [33], with adjustment for multiple testing (BH-*P*_adj._ < 0.05) [27].

Gene Ontology (GO) [34] and Kyoto Encyclopedia of Genes and Genomes (KEGG) [35] pathway analyses were applied to the significant gene sets, including differentially expressed gene (DEG) sets. These analyses identified and classified relevant biological processes, molecular functions, and pathways associated with these gene sets using the WEB-based GEne SeT AnaLysis Toolkit 2024 (WebGestalt 2024) [36].

After identifying significantly enriched GO terms, REVIGO [37] was used to reduce redundancy and summarize the results. Specifically, the list of significantly enriched GO terms (BH-*P*_adj._ < 0.05) was uploaded to the REVIGO web server, where the terms were clustered based on semantic similarity. The allowed similarity threshold was set to 0.7, and the resulting non-redundant GO terms were visualized to provide a clearer interpretation of the data.

### Enrichment analysis of QTL and GWAS data

Cattle QTL data were used to explore the genetic regulation of bovine mastitis-related traits. Specifically, we focused on QTLs associated with clinical mastitis, somatic cell count (SCC), and somatic cell score (SCS). Relevant QTLs were retrieved from the Cattle QTL Database, which provides comprehensive information on bovine QTLs, including their chromosomal locations [38]. To identify potential associations between these QTLs and bta-miR-223-related loci, we extracted the genomic coordinates of QTLs linked to mastitis-related traits and overlapped these with the genomic locations of bta-miR-223 target genes, as well as other related gene sets, including DEGs and putative target genes.

To further explore the relationship between bta-miR-223 and bovine mastitis, genome-wide association study (GWAS) data were leveraged. The GWAS data, obtained from analyses of 44 complex traits related to body type, reproduction, production, and health in a cohort of 27,214 USA Holstein bulls, provided summary statistics that included SNPs, their associated *P*-values, and effect sizes [39, 40]. The GWAS signal enrichment analysis was conducted using a sequence-based approach. GWAS SNPs were mapped to genes based on their genomic coordinates, without any additional physical distances or intervals (0 kb) from gene regions included in the analysis. The statistically significant GWAS SNPs were then used to determine whether bta-miR-223 related gene sets, such as DEGs or target genes, were enriched for GWAS signals. To assess the enrichment, a hypergeometric test [41] was performed using the hyperGSet function and a custom R script. The input for this test included the *P*-values from the GWAS summary statistics, where SNPs with *P*-values below a threshold of 0.01 were considered significant. Gene sets related to bta-miR-223, such as DEGs or predicted target genes, were analyzed to determine whether they contained a higher number of significant SNPs than would be expected by random distribution. The hyperGSet function output provided *P*-values that indicated the statistical significance of the enrichment for each gene set. The results of the hypergeometric test were adjusted for multiple comparisons using an adjustment for multiple tests (BH-*P*_adj._ < 0.05) [27].

## Results

### Optimization of bta-miR-223 inhibitor and mimic concentrations and development of a *S. aureus*-induced MAC-T cell inflammation model

**Fig. 1:**
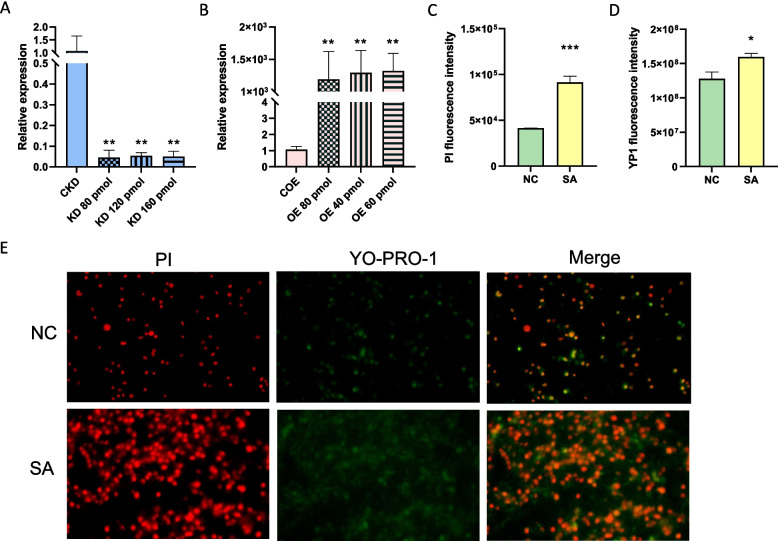
Evaluating bta-miR-223 modulation and *S. aureus* infection impact in MAC-T cells. Influence of various concentrations of inhibitors (**A**) and mimics (**B**) on bta-miR-223 expression levels. Effects of *S. aureus* challenge on PI (**C**) and YO-PRO-1 (**D**) fluorescence intensities in MAC-T cells. Statistical significance is denoted as follows: * denotes BH-*P*_adj._ < 0.05, ** denotes BH-*P*_adj._ < 0.01, *** denotes BH-*P*_adj._ < 0.001, when compared to the respective control groups. **E** Immunofluorescence depicting *S. aureus*-induced apoptosis and necrosis. NC (Negative Control): represents untreated cells. SA (*S. aureus*): represents cells treated with *S. aureus*. PI (propidium iodide): stains the necrotic cells in red; deeper red indicates higher necrosis. YO-PRO-1: stains apoptotic cells in green; deeper green indicates higher apoptosis. Merge: combines PI and YO-PRO-1 staining to show both necrosis and apoptosis; deeper colors indicate higher levels of cell death

Optimal concentrations of bta-miR-223 inhibitors achieved over 98% knockdown efficiency, significantly reducing bta-miR-223 expression compared to the control (Fig. 1A). In a parallel experiment, bta-miR-223 mimics led to a notable increase in bta-miR-223 levels, surpassing those in the control overexpression group (Fig. 1B).

Following infection with *S. aureus*, significant increases in both apoptosis and necrosis were observed in MAC-T cells, as demonstrated by elevated PI and YO-PRO-1 fluorescence intensity (Fig. 1C and D). Additionally, Fig. 1E shows immunofluorescence results comparing control (NC) and *S. aureus*-treated (SA) MAC-T cells. The control group showed minimal PI (red) and YO-PRO-1 (green) staining, indicating limited damage and apoptosis. In contrast, the treated group shows intense staining, indicating significant cell damage and apoptosis. The merged images confirm the successful creation of a *S. aureus*-induced MAC-T cell inflammation model.

### Protective effect of bta-miR-223 on *S. aureus*-induced apoptosis and necrosis in MAC-T cells

**Fig. 2:**
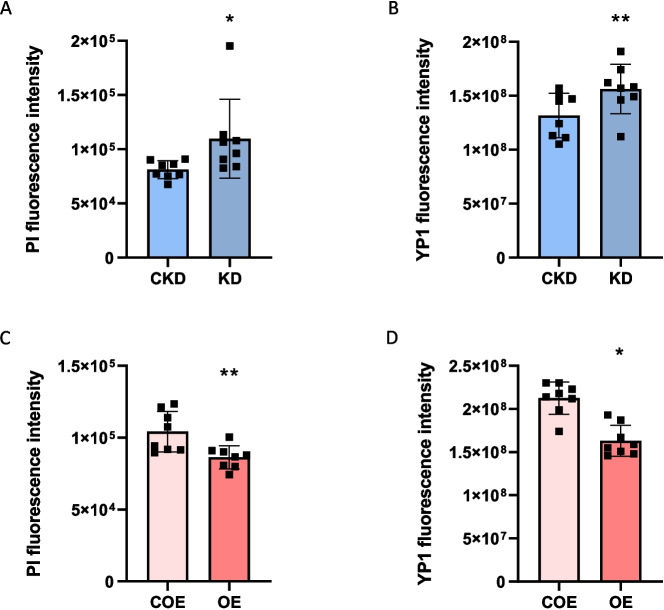
Role of bta-miR-223 in modulating cell death responses to *S. aureus* in MAC-T cells. Impact of bta-miR-223 knockdown on PI (**A**) and YO-PRO-1 (**B**). Fluorescence intensity in MAC-T cells after *S. aureus* infection. Protective effects of bta-miR-223 overexpression on PI (**C**) and YO-PRO-1 (**D**) fluorescence intensity in MAC-T cells exposed to *S. aureus*. CKD represents control knockdown, KD represents bta-miR-223 knockdown, COE represents control overexpression, and OE represents bta-miR-223 overexpression. Statistical significance is denoted as follows: * corresponds to BH-*P*_adj._ < 0.05, ** corresponds to BH-*P*_adj._ < 0.01, when compared to the respective control groups

Modulating bta-miR-223 expression significantly affected the levels of apoptosis and necrosis in MAC-T cells following infection with *S. aureus*. Specifically, knockdown of bta-miR-223 led to an increase in apoptosis and necrosis compared to control cells, indicating that reduced bta-miR-223 levels exacerbate cell death in response to bacterial infection (Fig. 2A and B). In contrast, overexpression of bta-miR-223 showed a protective role, resulting in a significant reduction of both apoptosis and necrosis (Fig. 2C and D). These findings demonstrate that bta-miR-223 modulation significantly influences cell death during *S. aureus* infection, suggesting a potential regulatory role in apoptotic and necrotic responses.

### Overview of RNA sequencing data on bta-miR-223 modulation and *S. aureus* challenge

**Fig. 3:**
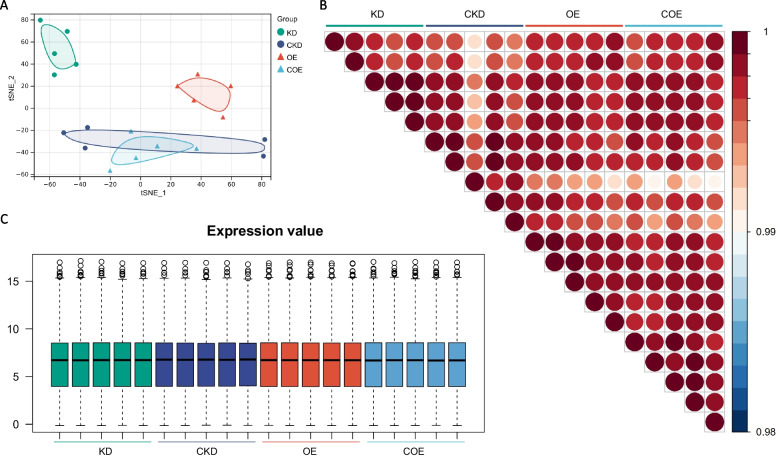
Transcriptomic features of bta-miR-223 knockdown and overexpression in MAC-T cells. **A** t-SNE clustering analysis: This analysis clusters samples from four experimental groups based on their transcriptional profiles (FPKM), each denoted by a distinct colour and symbol. **B** Correlation heatmap: This heatmap depicts pairwise similarities among all samples under the experimental conditions, with a colour gradient from dark blue to dark red indicating correlation coefficients ranging from 0.98 to 1.00. **C** Expression value distribution: The boxplot displays the distribution of expression values for each sample

The sequencing generated a mean of 43,456,787 clean sequencing reads per sample, with an average Q30 score of 92.92%, indicating high-quality data (Table S1). A t-SNE plot demonstrated effective clustering among samples from each group, with the exception of the CKD group (Fig. 3A). This clustering pattern suggests a high degree of consistency in mRNA expression characteristics within each group (Fig. 3B). No significant differences were observed in overall mRNA expression levels across the treatment groups (Fig. 3C). This result indicating that bta-miR-223 modulation did not induce broad transcriptomic changes but may instead influence specific pathways or gene sets.

### Identification of co-expression modules linked to inflammation and apoptosis pathways via WGCNA

**Fig. 4:**
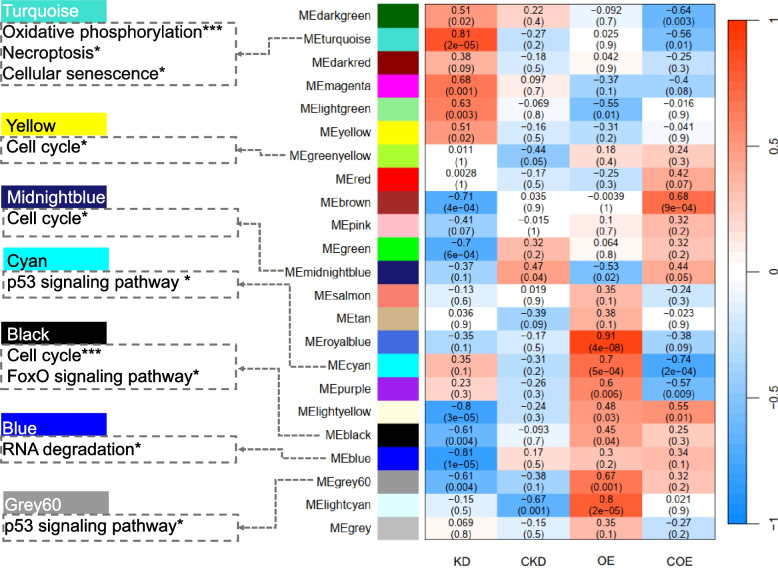
WGCNA of gene expression modules in response to bta-miR-223 modulation in MAC-T cells. This heatmap depicts the correlations between gene expression modules and the effects of bta-miR-223 modulation under various experimental conditions: KD (knockdown), CKD (control for knockdown), OE (overexpression), and COE (control for overexpression). Each row, represented by a unique colour, corresponds to a distinct gene module. The cells within the heatmap display correlation coefficients; *P*-values in parentheses indicate the statistical significance of the correlations between each module and the experimental groups (bta-miR-223 modulation). The intensity of the colours—red indicating positive and blue indicating negative correlations. To the left, coloured boxes corresponding to the modules highlight specific biological pathways affected by the treatments, identified through KEGG enrichment analysis. The significance levels of these pathways are indicated by BH-*P*_adj_., with significance levels noted (* BH-*P*_adj__._ < 0.05, ** BH*-P*_adj__. _< 0.01, *** BH*-P*_adj._ < 0.001)

The top 80% of highly variable genes were subjected to WGCNA analysis, resulting in the identification of 23 modules, among which 22 were co-expression modules and one was an unclustered module. KEGG pathway enrichment analysis was conducted on the genes in modules that were significant correlations with the treatment groups. The analysis revealed that genes within the modules Turquoise, Yellow, Green, and Grey60 were significantly enriched in pathways related to inflammation, such as the FoxO signalling pathway, the p53 signalling pathway, and necroptosis (BH-*P*_adj._ < 0.05, Fig. 4). The turquoise module was found to be enriched in pathways related to necroptosis, the cell cycle, and cellular senescence, indicating its involvement in the regulation of cell cycle processes. Notably, the genes within the Grey60 module were significantly enriched in the p53 signalling pathway, displaying a significant negative correlation with the knockdown group and a positive correlation with the overexpression treatment group. These results indicate that bta-miR-223 plays a crucial role in apoptosis, necrosis, and immune regulation processes of MAC-T cells following *S. aureus* infection.

### Bta-miR-223 modulates key pathways by activating cell cycle activity while reducing apoptotic responses

**Fig. 5:**
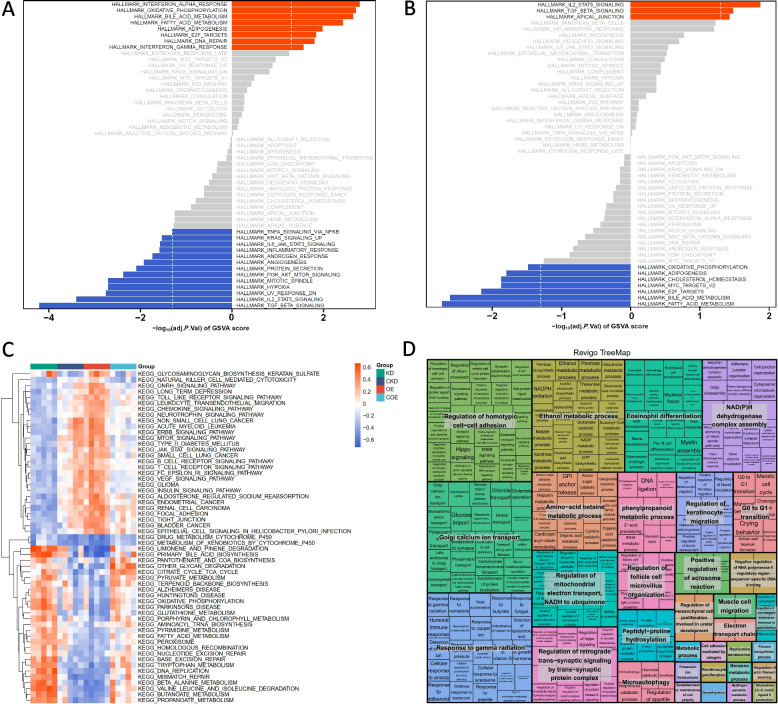
GSVA pathway enrichment analysis of bta-miR-223-modulated MAC-T cells. **A** Bar graph of GSVA enrichment scores for hallmark pathways in the KD vs. CKD comparison. Pathways significantly enriched are displayed with positive enrichment scores in orange and negative enrichment scores in blue. **B** Bar graph of GSVA enrichment scores for hallmark pathways in the OE vs. COE comparison. **C** Heatmap showing the enrichment of KEGG pathways in KD, CKD, OE, and COE groups in *S. aureus*-infected MAC-T cells. **D** REVIGO TreeMap visualization of shared Biological Process (BP) GO terms enriched in the KD vs. CKD and OE vs. COE comparisons. Each rectangle represents a GO term, with related terms grouped together. The size of the rectangles corresponds to the significance of the enrichment, with colours indicating different biological processes

The GSVA analysis highlighted several significant findings. For the hallmark gene sets, pathways such as E2F targets, IL2 STAT5 signalling, TGF beta signalling, and oxidative phosphorylation showed significant enrichment in the bta-miR-223 KD and OE groups compared to their respective controls (CKD and COE), with opposing trends (Fig. S1). Specifically, the KD vs. CKD comparison revealed increased enrichment in IL2 STAT5 signalling and TGF beta signalling, indicating heightened immune and apoptotic responses (Fig. 5A, Table S2). In contrast to this, the OE vs. COE comparison was more enriched in E2F targets and oxidative phosphorylation, suggesting enhanced cell cycle activity and metabolic processes (Fig. 5B, Table S3). Similarly, in the KEGG pathway analysis, we observed comparable trends. For example, pathways related to immune responses, such as the JAK-STAT signalling pathway and Toll-like receptor signalling pathway, were significantly enriched in the KD vs. CKD comparison, reflecting elevated immune activity and increased apoptosis and necrosis (Fig. 5C, Table S4). Conversely, the mTOR signalling pathway was more enriched in the OE vs. COE comparison, corresponding to enhanced cellular growth, reduced apoptosis, and necrosis (Fig. 5C, Table S5). In the GO analysis, terms related to the immune response and apoptosis, such as antibacterial humoral response and response to interleukin-6, were significantly enriched in the KD vs. CKD comparison, while GO terms like regulation of glucose import were more enriched in the OE vs. COE comparison (Fig. 5D, Table S6, Table S7). Overall, these GSVA results demonstrate that bta-miR-223 modulates key pathways in *S. aureus*-infected MAC-T cells by enhancing cell cycle processes while reducing apoptotic responses.

### Identification and functional annotation of differentially expressed genes in *S. aureus*-infected MAC-T cells

**Fig. 6:**
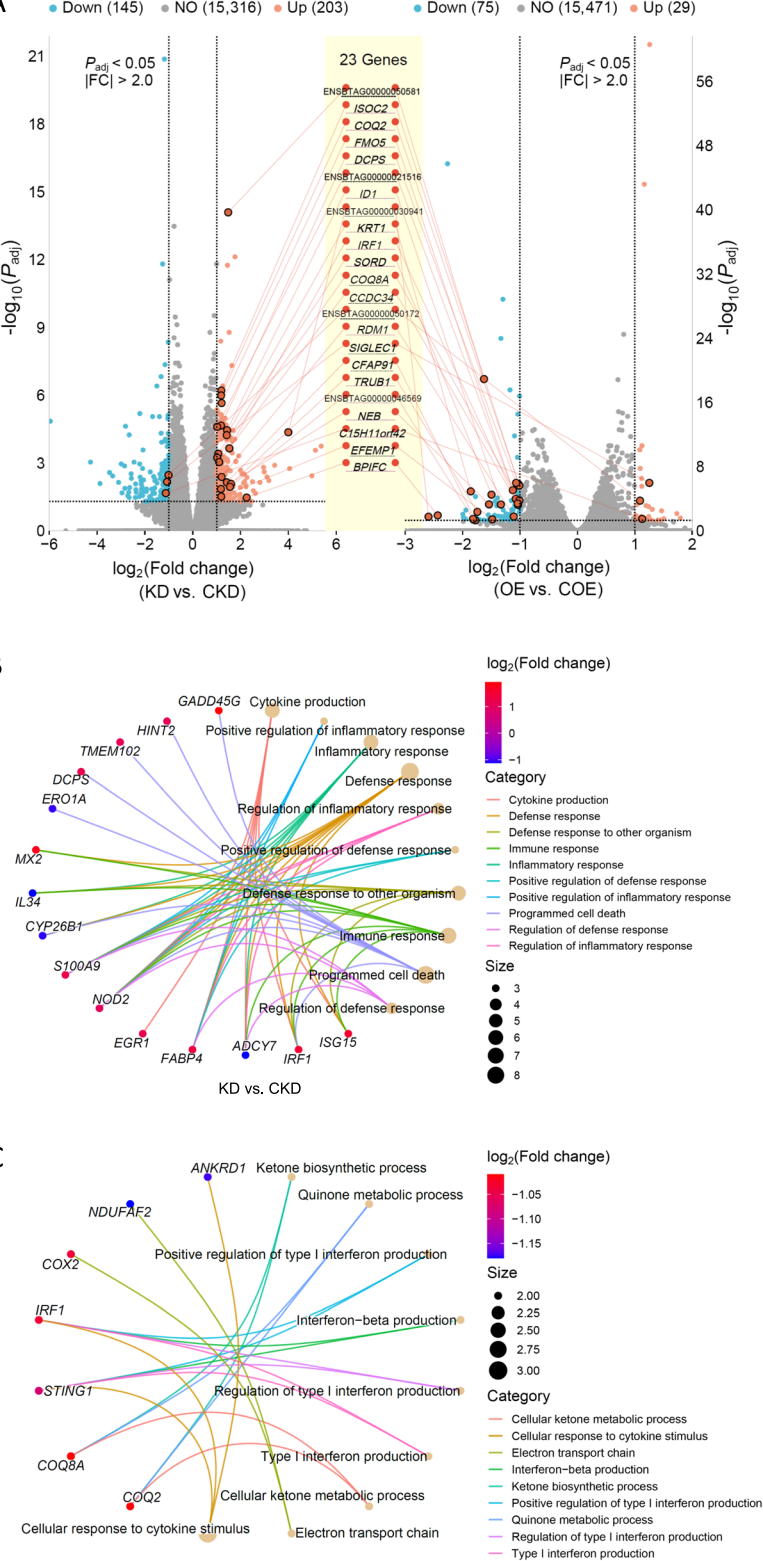
Differential gene expression and functional enrichment in bta-miR-223-modulated MAC-T cells. **A** Volcano plots showing differentially expressed genes (DEGs) in the bta-miR-223 KD vs. CKD and OE vs. COE comparisons for *S. aureus*-infected MAC-T cells. Significant DEGs (BH-*P*_adj._ < 0.05) are indicated with coloured dots: blue for downregulated genes and red for upregulated genes. The 23 genes consistently differentially expressed across both conditions are highlighted in the centre. **B** GO enrichment analysis of DEGs in the KD vs. CKD comparison. The network shows significantly enriched GO terms related to immune response and inflammation. **C** GO enrichment analysis of DEGs in the OE vs. COE comparison. The network shows significantly enriched GO terms

We found 203 genes upregulated and 145 genes downregulated in the KD vs. CKD comparison (BH-*P*_adj._ < 0.05, Table S8). In the OE vs. COE comparison, 29 genes were upregulated, and 75 genes were downregulated (BH-*P*_adj._ < 0.05, Table S9). Among these, 23 genes were consistently differentially expressed, with genes upregulated in KD vs. CKD comparisons being downregulated in OE vs. COE comparisons, and vice versa (Fig. 6A).

The results showed that the DEGs in the KD vs. CKD comparison were significantly enriched in the ubiquinone and other terpenoid-quinone biosynthesis pathway. GO enrichment analysis indicated that the DEGs in the KD group were significantly enriched in immune-related GO terms, such as antibacterial humoral response and response to interleukin-6 (Fig. 6B). The DEGs in the OE vs. COE comparison produced similar results (Fig. 6C).

### Identification of key candidate target genes of bta-miR-223 in *S. aureus*-infected MAC-T cells

**Fig. 7:**
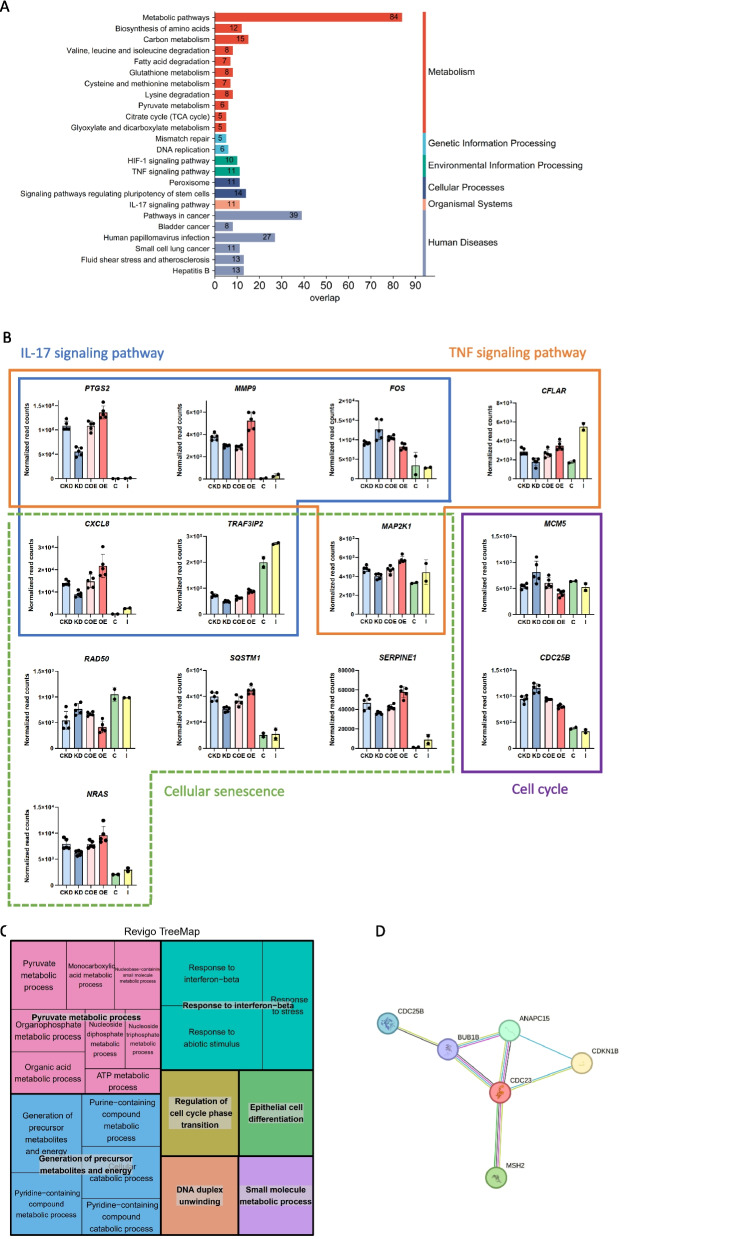
Functional analysis of potential bta-miR-223 target genes in *S. aureus*-infected MAC-T cells. **A** KEGG pathway enrichment analysis: the bar graph shows the KEGG pathway enrichment analysis of the 693 potential target genes of bta-miR-223 (BH-*P*_adj._ < 0.05). **B** Differential gene expression analysis: the bar plots display the expression levels of key genes (normalized read counts) in critical pathways. Comparisons are shown between the knockdown (KD), overexpression (OE), *S. aureus* infection group (I), and their respective control groups. The *S. aureus* infection group (I) and control group (C) are derived from a study on *S. aureus* infection in different regions of the mammary gland of lactating cows [17]. **C** GO Analysis—REVIGO TreeMap: the tree map visualizes the Gene Ontology (GO) terms enriched among the potential target genes, focusing on the regulation of cell cycle phase transition, response to stimuli, and epithelial cell differentiation. The size and colour of the boxes represents the significance and categorization of the terms, respectively. **D** Gene interaction network: the network diagram depicts significant interactions among proteins involved in regulating cell cycle phase transition; highlighted proteins include CDC25B, CDC23, ANAPC15, CDKN1B, MSH2, and BUB1B

To further understand the function of bta-miR-223, we expanded the gene screening criterion (BH-*P*_adj._ < 0.05) to select genes exhibiting opposing trends in the KD and OE comparisons. Through analysis, we identified 693 common potential target genes of bta-miR-223 (Table S10). KEGG pathway enrichment analysis of this gene set revealed significant enrichment in several pathways, particularly metabolic pathways, and the TNF signalling pathway and the IL-17 signalling pathway (Fig. 7A, Table S11).

In the cell cycle pathway, genes such as *MCM5* and *CDC25B*, and in the TNF signalling pathway, genes like *FOS*, were significantly upregulated in the KD group while significantly downregulated in the OE group (Fig. 7B). Moreover, genes like *PTGS2* and *MMP9* in the IL-17 signalling pathway and *CXCL8* and *TRAF1* in the TNF signalling pathway showed significant downregulation in the KD group and significant upregulation in the OE group (Fig. 7B). Additionally, as shown in Fig. 7C, GO analysis indicated significant interactions among genes involved in the regulation of cell cycle phase transition. Specifically, significant interactions were identified between proteins such as CDC25B and CDC23 (Table S12, Fig. 7D).

We identified 1,279 gene transcripts corresponding to 589 individual gene targets of bta-miR-223 (Table S13). By intersecting these with the previously identified potential target genes, we identified 28 robust potential target genes, further supporting the regulatory function of bta-miR-223. As shown in Fig. 7B, these genes are involved in key cell cycle and immune-related pathways. Furthermore, the present study showed that the expression trends of certain genes were consistent with bta-miR-223 target genes identified in our previous study on *S. aureus* infection in different regions of the mammary gland of individual cows (Fig. 7B) [17].

### Bta-miR-223’s potential target genes significantly enriched in mastitis-related QTLs and SNPs

**Fig. 8:**
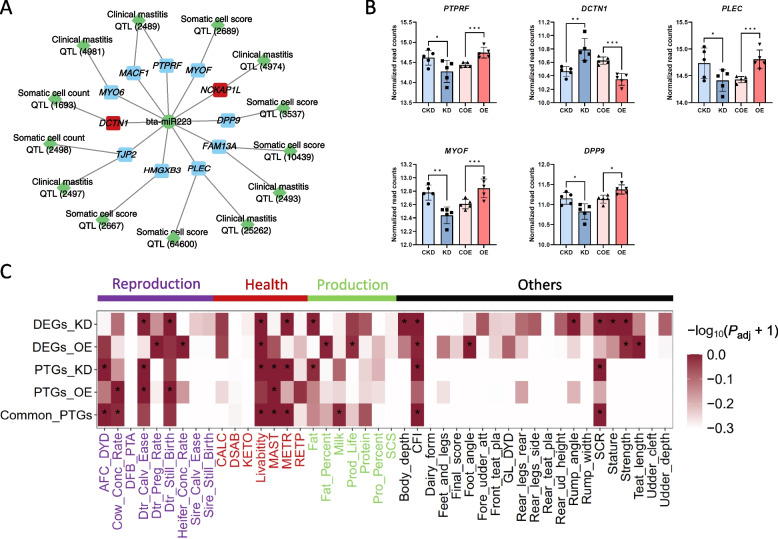
Functional analysis of bta-miR-223 and its potential target genes in dairy cattle. **A** The regulatory relationships between bta-miR-223 and its robust potential target genes. Each gene is linked to specific QTLs associated with somatic cell count (SCC), somatic cell score (SCS), and clinical mastitis traits. **B** The normalized read counts of key genes (*PTPRF*, *DCTN1*, *PLEC*, *MYOF*, and *DPP9*) in control (CKD, COE), knockdown (KD), and overexpression (OE) groups. Significant changes in expression levels are indicated by asterisks (^*^BH-*P*_adj._ < 0.05, ^**^BH-*P*_adj._ < 0.01, ^***^BH-*P*_adj._ < 0.001). **C** The heatmap shows the enrichment of gene sets in various reproduction, health, and production traits. Gene sets analysed include DEGs_KD, DEGs_OE, PTGs_KD, PTGs_OE, and Common_PTGs. Traits are categorized as reproduction (purple), health (red), production (green), and others (black). The color intensity represents the −log_10_(*P*_adj._ + 1) value, indicating the level of significance of the enrichment. Asterisks within the heatmap cells denote significant enrichment

In the KD vs. CKD comparison, 154 genes overlapped with 80 QTLs related to SCC/SCS and clinical mastitis traits (Table S14). For the OE vs. COE comparison, 20 genes were found to overlap with 19 QTLs (Table S15). In the potential target gene set, 298 genes overlapped with 118 QTLs related to SCC/SCS and clinical mastitis traits (Table S16). The regulatory relationships between robust potential target genes of bta-miR-223 and QTLs for SCC/SCS and clinical mastitis traits are illustrated in Fig. 8A. Notably, the expression levels of key genes (*PTPRF*, *DCTN1*, *PLEC*, *MYOF*, and *DPP9*) in the control (CKD, COE), KD, and OE groups showed significant changes, underscoring their potential roles in regulating SCC/SCS and clinical mastitis (Fig. 8B).

This study used signal enrichment of GWAS data from Holstein bulls (*n* = 27,143) by mapping SNPs to genes based on their genomic coordinates and evaluating the enrichment of bta-miR-223-related gene sets using a hypergeometric test. The results revealed significant enrichment in traits such as mastitis (MAST) (Fig. 8C). Interestingly, these gene sets were also significantly enriched in production traits such as milk yield and fat percentage, as well as longevity traits (Table S17). This suggests that bta-miR-223, in addition to being a key miRNA associated with mastitis resistance, may also influence production traits.

## Discussion

In this study, we demonstrated that elevated expression of miR-223 leads to reduced apoptosis and necrosis in mammary epithelial cells. This finding aligns with previous research in various mammalian species and tissues and emphasises the importance of miR-223 in cellular protection mechanisms [42–44]. For example, in human hepatocytes, miR-223 has been reported to inhibit apoptosis by targeting the *FOXO3* gene, which is involved in the regulation of cell death pathways [45]. Similarly, in murine models, miR-223 has been implicated in the protection of cardiac myocytes from apoptosis induced by ischemic injury by targeting multiple pro-apoptotic genes [14]. Previous studies have indicated that miR-223 plays a critical role in the regulation of mammary epithelial cell function. For instance, research showed that miR-223 suppresses apoptosis in mammary epithelial cells by targeting the *NLRP3* inflammasome pathway, thereby reducing inflammation and promoting cell survival. In the context of dairy cows, miR-223 has been identified as a key regulator in the immune response to mastitis [46]. In 2019, Cai and colleagues performed miRNA expression profiling of bovine mammary glands infected with *S. aureus* and found that miR-223 was significantly upregulated in response to infection [47]. This upregulation is believed to play a protective role by modulating inflammatory responses and reducing cell death in mammary tissues. The protective role of miR-223 is not limited to a specific type of tissue. Additionally, in the context of lung epithelial cells, miR-223 has been shown to mitigate inflammation and cell death, suggesting its broad protective role across different epithelial cell types [48]. These findings collectively underscore the potential of miR-223 as a therapeutic target in various inflammatory and injury contexts.

The results from our GSVA analysis provide valuable insights into the role of bta-miR-223 in modulating cellular pathways related to apoptosis, necrosis, and immune responses in the context of *S. aureus* infection and mastitis. Our findings indicate that miR-223 overexpression enhances pathways involved in cell cycle progression and metabolic processes, such as E2F targets and oxidative phosphorylation, while simultaneously reducing pathways associated with immune and apoptotic responses. Conversely, miR-223 knockdown results in the upregulation of immune response pathways, including IL2‒STAT5 signalling and TGF beta signalling, which are associated with increased cell apoptosis and necrosis. This differential enrichment suggests that miR-223 acts as a critical regulator in maintaining cellular homeostasis during infection. By promoting pathways that enhance cell survival and metabolic activity while suppressing inflammatory and apoptotic pathways, miR-223 overexpression may offer a protective mechanism against *S. aureus*-induced cell damage. This is consistent with previous studies demonstrating the anti-apoptotic and anti-inflammatory roles of miR-223 in various cell types [49–51]. The GO analysis revealed that terms such as calcium-activated phospholipid scrambling, immune complex clearance, antibacterial humoral response, and response to interleukin-6 were significantly enriched in the KD vs. CKD comparison. These enrichments suggest that miR-223 knockdown enhances the cellular immune response, which may help in clearing bacterial infections and mitigating inflammation [46, 52].

The identification of key candidate target genes for miR-223 in *S. aureus*-infected MAC-T cells offers profound insights into the molecular mechanisms underlying bovine mastitis. Specifically, genes such as *CDC25B*, *CDC23*, *ANAPC15*, and *CDKN1B* play crucial roles in cell cycle regulation and immune response, aligning well with the results of this study. The *CDC25B* gene encodes an essential regulator of the cell cycle [53], which is a phosphatase that activates cyclin-dependent kinases (CDKs), promoting the transition from G2 to M phase [54]. In this study, the significant upregulation of *CDC25B* in the KD group and its downregulation in the OE group highlight its pivotal role in cell cycle progression influenced by miR-223. Similarly, results for the *ANAPC15* [55] and *CDKN1B* [56] genes further corroborate the importance of cell cycle regulation. The ANAPC15 protein is part of the APC/C complex, crucial for the regulation of cell division. The *ANAPC15* expression pattern in our study suggests it is a significant target of miR-223 in controlling cell cycle transitions. CDKN1B, a well-understood cyclin-dependent kinase inhibitor, regulates cell cycle progression at G1 by inhibiting CDK activity [57]. The differential expression of *CDKN1B* in the KD and OE groups supports the role of miR-223 in cell cycle arrest and proliferation. In summary, the differential expression of *CDC25B*, *ANAPC15*, and *CDKN1B* in response to miR-223 manipulation underscores their crucial roles in cell cycle regulation during *S. aureus* infection. These findings provide a robust framework for further functional studies and potential therapeutic strategies targeting miR-223 in bovine mastitis.

The exploration of the role of miR-223 in *S. aureus*-induced mastitis reveals its significant influence on key genes associated with this disease. The identification of DEGs and potential target genes, particularly *PTPRF*, *DCTN1*, *PLEC*, *MYOF*, and *DPP9*, underscores the critical role of miR-223 in modulating mastitis-related traits in dairy cattle. The *PTPRF* gene encodes a critical regulator of cell signalling pathways involved in immune responses [58]. The significant expression changes observed for *PTPRF* in the KD and OE groups indicate that miR-223 may regulate mastitis resistance by modulating the activity of this gene. This modulation could affect cellular adhesion and migration, crucial processes in the immune response to infection [59]. The *DCTN1* [60], *MYOF* [61], and *DPP9* [62] genes encode key regulators of intracellular transport, membrane repair, and protein degradation, respectively, and their expression changes driven by miR-223 suggest a comprehensive modulation of immune response and cellular resilience during mastitis. The enrichment of these genes in QTLs associated with SCC, SCS, and clinical mastitis traits further underscores their importance. The integration of GWAS data from Holstein bulls reveals that miR-223 not only plays a pivotal role in mastitis resistance but also potentially influences production traits such as milk yield, milk fat percentage, and longevity traits. This dual impact suggests a broader regulatory function of miR-223 in dairy cattle, bridging immune response and production efficiency.

## Conclusion

In summary, our study demonstrates the vital protective role of miR-223 in mammary epithelial cells during *S. aureus* infection. By regulating critical genes such as *PTPRF*, *DCTN1*, and *DPP9*, miR-223 emerges as a key molecular modulator that balances cellular processes, contributing to mastitis resistance and production traits like milk yield and fat content. These findings align with the hypothesis that miR-223 acts as a dual regulator of disease resistance and production efficiency, offering new insights into its multifunctional role. Importantly, this study identifies miR-223 as a promising target for genetic improvement strategies aimed at mitigating mastitis and enhancing dairy cattle performance. Future research should focus on investigating the in vivo impact of miR-223, which will be crucial for translating these findings into effective therapeutic and genetic improvement strategies.

## Supplementary Information

Additional file 1: Table S1. Summary of sequence reads. Table S2. KD vs. CKD in hallmark gene sets by GSVA analysis. Table S3. OE vs. COE in hallmark gene sets by GSVA analysis. Table S4. KD vs. CKD in KEGG gene sets by GSVA analysis. Table S5. OE vs. COE in KEGG gene sets by GSVA analysis. Table S6. KD vs. CKD in GO gene sets by GSVA analysis. Table S7. OE vs. COE in GO-BP gene sets by GSVA analysis. Table S8. The differentially expressed genes in KD vs. CKD. Table S9. The differentially expressed genes in OE vs. COE. Table S10. Potential target genes. Table S11. KEGG pathway enrichment of common genes in KD vs. CKD and OE vs. COE comparisons. Table S12. GO enrichment of common genes in KD vs. CKD and OE vs. COE comparisons. Table S13. Target genes of bta-miR-223. Table S14. QTL enrichment results for differentially expressed genes in KD vs. CKD. Table S15. QTL enrichment results for differentially expressed genes in OE vs. COE. Table S16. QTL enrichment results for potential target genes. Table S17. The relationships between 44 complex traits and five gene sets.

Additional file 2: Fig. S1. GSVA pathway enrichment analysis of bta-miR-223-modulated MAC-T cells.

## Data Availability

The datasets generated and/or analysed during the current study are available from the corresponding author on reasonable request.

## Abbreviations

BAM: Binary alignment/map format
BH-*P*_adj._: Benjamini–hochberg adjusted *P*-value
Bta-miR-223: *Bos tauru**s* miRNA-223
CKD: Control knockdown
COE: Control overexpression
DEG: Differentially expressed gene
DMEM: Dulbecco’s modified eagle medium
FBS: Foetal bovine serum
GO: Gene Ontology
GSVA: Gene set variation analysis
GWAS: Genome-wide association study
KD: Knockdown
KEGG: Kyoto Encyclopedia of Genes and Genomes
MAC-T: Mammary alveolar cell-T
MiRNA: MicroRNA
MOI: Multiplicity of infection
OE: Overexpression
Opti-MEM: Optimized minimum essential medium
PBS: Phosphate-buffered saline
PI: Propidium iodide
qPCR: Quantitative polymerase chain reaction
QTL: Quantitative trait loci
RNA-seq: RNA sequencing
SAM: Sequence alignment/map format
SCC: Somatic cell count
SCS: Somatic cell score
*S. aureus*: *Staphylococcus aureus*
SNP: Single nucleotide polymorphism
t-SNE: T-distributed stochastic neighbor embedding
TSB: Tryptic soy broth
WGCNA: Weighted gene co-expression network analysis
YP1: YO-PRO-1
