# Supplementary figures and images for: Functional validation to explore the protective role of miR-223 in Staphylococcus aureus-induced bovine mastitis
Source: J Anim Sci Biotechnol. 2025 Mar 4;16:34. doi: 10.1186/s40104-025-01152-6 (PMC11877765; doi:10.1186/s40104-025-01152-6)

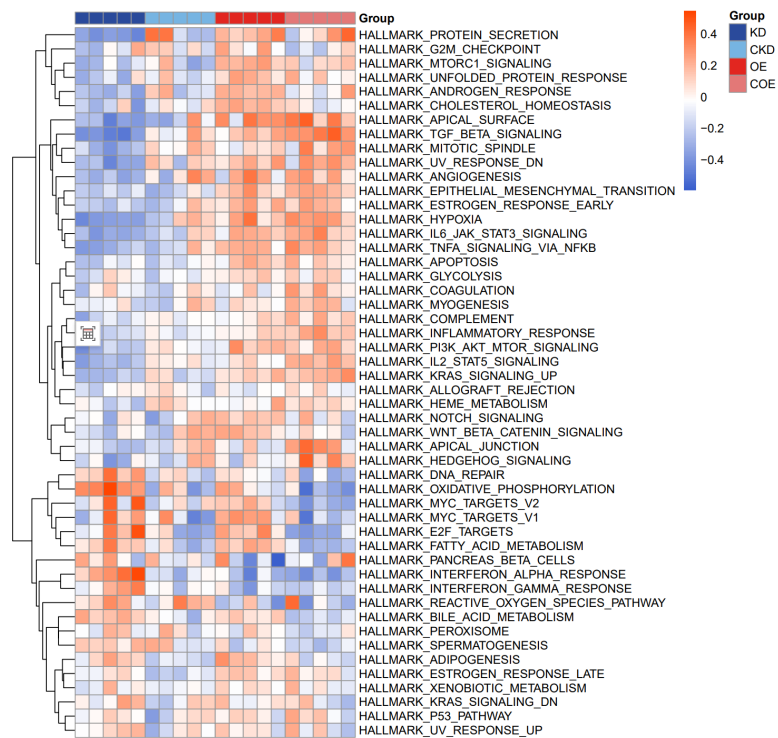

Supplement: Supplementary file 2 — Additional file 2: Fig. S1. GSVA pathway enrichment analysis of bta-miR-223-modulated MAC-T cells. [file 40104_2025_1152_MOESM2_ESM.pdf]
